# Engineering of a miniaturized, robotic clinical laboratory

**DOI:** 10.1002/btm2.10084

**Published:** 2018-01-19

**Authors:** Marilyn B. Nourse, Kate Engel, Samartha G. Anekal, Jocelyn A. Bailey, Pradeep Bhatta, Devayani P. Bhave, Shekar Chandrasekaran, Yutao Chen, Steven Chow, Ushati Das, Erez Galil, Xinwei Gong, Steven F. Gessert, Kevin D. Ha, Ran Hu, Laura Hyland, Arvind Jammalamadaka, Karthik Jayasurya, Timothy M. Kemp, Andrew N. Kim, Lucie S. Lee, Yang Lily Liu, Alphonso Nguyen, Jared O'Leary, Chinmay H. Pangarkar, Paul J. Patel, Ken Quon, Pradeep L. Ramachandran, Amy R. Rappaport, Joy Roy, Jerald F. Sapida, Nikolay V. Sergeev, Chandan Shee, Renuka Shenoy, Sharada Sivaraman, Bernardo Sosa‐Padilla, Lorraine Tran, Amanda Trent, Thomas C. Waggoner, Dariusz Wodziak, Amy Yuan, Peter Zhao, Daniel L. Young, Channing R. Robertson, Elizabeth A. Holmes

**Affiliations:** ^1^ Assay Development Theranos, 7373 Gateway Boulevard Newark CA 94560; ^2^ Systems Integration Theranos, 7373 Gateway Boulevard Newark CA 94560; ^3^ Computational Biosciences Theranos, 7373 Gateway Boulevard Newark CA 94560; ^4^ Software Development Theranos, 7373 Gateway Boulevard Newark CA 94560; ^5^ Engineering Theranos, 7373 Gateway Boulevard Newark CA 94560; ^6^ Reagent Development Theranos, 7373 Gateway Boulevard Newark CA 94560; ^7^ Theranos, 7373 Gateway Boulevard Newark CA 94560

**Keywords:** automation, clinical chemistry, diagnostics, hematology, immunoassay, laboratory testing, molecular diagnostics

## Abstract

The ability to perform laboratory testing near the patient and with smaller blood volumes would benefit patients and physicians alike. We describe our design of a miniaturized clinical laboratory system with three components: a hardware platform (ie, the miniLab) that performs preanalytical and analytical processing steps using miniaturized sample manipulation and detection modules, an assay‐configurable cartridge that provides consumable materials and assay reagents, and a server that communicates bidirectionally with the miniLab to manage assay‐specific protocols and analyze, store, and report results (i.e., the virtual analyzer). The miniLab can detect analytes in blood using multiple methods, including molecular diagnostics, immunoassays, clinical chemistry, and hematology. Analytical performance results show that our qualitative Zika virus assay has a limit of detection of 55 genomic copies/ml. For our anti‐herpes simplex virus type 2 immunoglobulin G, lipid panel, and lymphocyte subset panel assays, the miniLab has low imprecision, and method comparison results agree well with those from the United States Food and Drug Administration‐cleared devices. With its small footprint and versatility, the miniLab has the potential to provide testing of a range of analytes in decentralized locations.

## 1 INTRODUCTION

Clinical laboratory tests are invaluable in diagnosing and managing diseases.^1^, ^2^ Currently, such tests are most often conducted in centralized laboratories using a variety of analytical methods executed on a multitude of large‐scale analyzers.^3^, ^4^ These tests can also require clinically important amounts of blood,^5^, ^6^, ^7^, ^8^, ^9^, ^10^, ^11^, ^12^, ^13^ sometimes including oversampling volumes^11^, ^13^, ^14^ and often involve complex pre‐analytical and analytical processing subject to human error.^15^ In addition, samples must be transported from the patient to the laboratory, a process that may compromise sample stability and requires tracking procedures to avoid losing, mislabeling, or mishandling samples.^15^

The ability to conduct a wide variety of tests near the patient on smaller volumes of blood especially benefits neonates,^16^ the elderly,^17^, ^18^ those who need frequent testing,^12^ and those with fragile or difficult to access veins. The decreased chance of losing or mislabeling samples helps physicians and hospitals provide better care.^5^, ^6^, ^7^, ^8^, ^9^, ^10^ Testing at the point of care is more convenient for the patient and increases patient compliance in fulfilling laboratory test orders.^19^, ^20^, ^21^ Currently available point of care systems have made progress toward multiplexing commonly ordered tests,^22^, ^23^, ^24^, ^25^, ^26^, ^27^, ^28^ but sometimes suffer from performance limitations when compared to centralized laboratories.^23^ In addition, many point of care analyzers lack connectivity that would allow for comprehensive oversight and tracking of system performance. When implemented with the same analytical performance specifications and relevant test menu as centralized laboratories, a connected, decentralized bench top testing platform could potentially augment standard clinical laboratory services.^29^

We describe a simple‐to‐use miniaturized clinical laboratory system designed to test a diverse range of clinical analytes in distributed laboratory or near‐patient settings. The system includes a hardware platform that performs the tests (i.e., the miniLab), an assay‐configurable cartridge containing all necessary consumable assay materials, and a server that communicates bidirectionally with the miniLab to manage assay‐specific instructions and results (i.e., the virtual analyzer). We demonstrate the functionality of the system with analytical performance metrics across a variety of analyte classes, as represented by a Zika virus^30^, ^31^ molecular diagnostic assay, an anti‐herpes simplex virus type 2 (HSV‐2) immunoglobulin G (IgG) immunoassay, a clinical chemistry lipid panel, and a lymphocyte subset hematology panel. The Theranos technologies or assays described here have not been cleared, approved, or authorized for diagnostic use by the United States Food and Drug Administration (FDA).

## 2 RESULTS

### 2.1 System design

**Figure 1:**
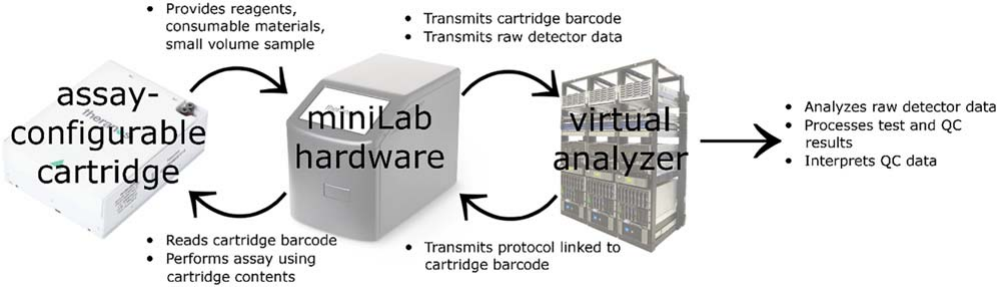
Miniaturized clinical laboratory system overview. Materials and information interface and exchange between the cartridge, the miniLab, and the virtual analyzer. Abbreviation: QC = quality control

The disposable assay‐configurable cartridge, which contains the blood sample and all reagents and consumable vessels required to conduct one or more analytical tests, inserts into the miniLab hardware platform (Figure 1, Supporting Information Video 1). The miniLab reads the barcode on the cartridge and sends it to the virtual analyzer server, which, in return, transmits the cartridge‐specific protocol to the miniLab. The miniLab uses its robotic sample processing modules and cartridge contents to perform step‐by‐step specimen and reagent handling procedures as defined by the assay protocol. Following assay execution, the miniLab sends the protocol status and raw detector outputs to the virtual analyzer. The virtual analyzer analyzes the raw data and processes it into assay results for the sample and any controls. The virtual analyzer may also be used to communicate sample or quality control (QC) results for review and oversight by a laboratorian. This system design aims to allow the miniLab to operate in decentralized laboratory settings yet maintain many key qualities common to a centralized clinical laboratory.

Designing a laboratory testing platform that has the capability to measure multiple analyte classes, yet is simple enough to use by operators with minimal prior laboratory testing experience and compact enough to install on a countertop presented numerous challenges. To achieve the flexibility necessary to perform myriad clinical laboratory tests as well as maintain compatibility with potential future tests, we designed the platform to use miniaturized reagent and reaction vessels combined with a compact, versatile material‐handling robot that has both pick‐and‐place and liquid‐handling capabilities. The design required a mechanism to process anticoagulated whole blood to plasma and multiple detector modules to measure various analyte classes. The material‐handling robot needed to accurately and precisely aspirate and dispense microliter volumes of liquids with differing rheological properties, mix fluids, resuspend particles, and transport vessels and cuvettes to different areas of the platform. Additionally, the entire platform required stable temperature control despite air exchange with variable ambient temperatures and filtering to provide protection for personnel and the environment. All of these modular elements had to be miniaturized and optimized to fit and function reliably together in a chassis with a volume similar to that of a desktop laser printer. Furthermore, careful hardware, firmware, and software integration was required to provide a comprehensive architecture for the successful operation of the miniLab while simultaneously facilitating ease‐of‐use.

We sought to limit the scope of system maintenance such as cleaning the device or refreshing reagents. Thus, we designed the assay‐specific cartridge to contain all necessary reagents and consumable materials for each assay and to prevent the miniLab hardware from making direct contact with the sample or other liquids. The cartridge housing required fixed dimensions and configuration to interface with the miniLab, yet also necessitated a flexible layout to accommodate a broad combination of specialized vessels and reagents needed for varied test menus. The cartridge lid needed to open inside the miniLab and close again upon ejection to protect the contents and prevent exposure of the user to potentially hazardous materials.

### 2.2 System architecture

#### 2.2.1 The miniLab

**Figure 2:**
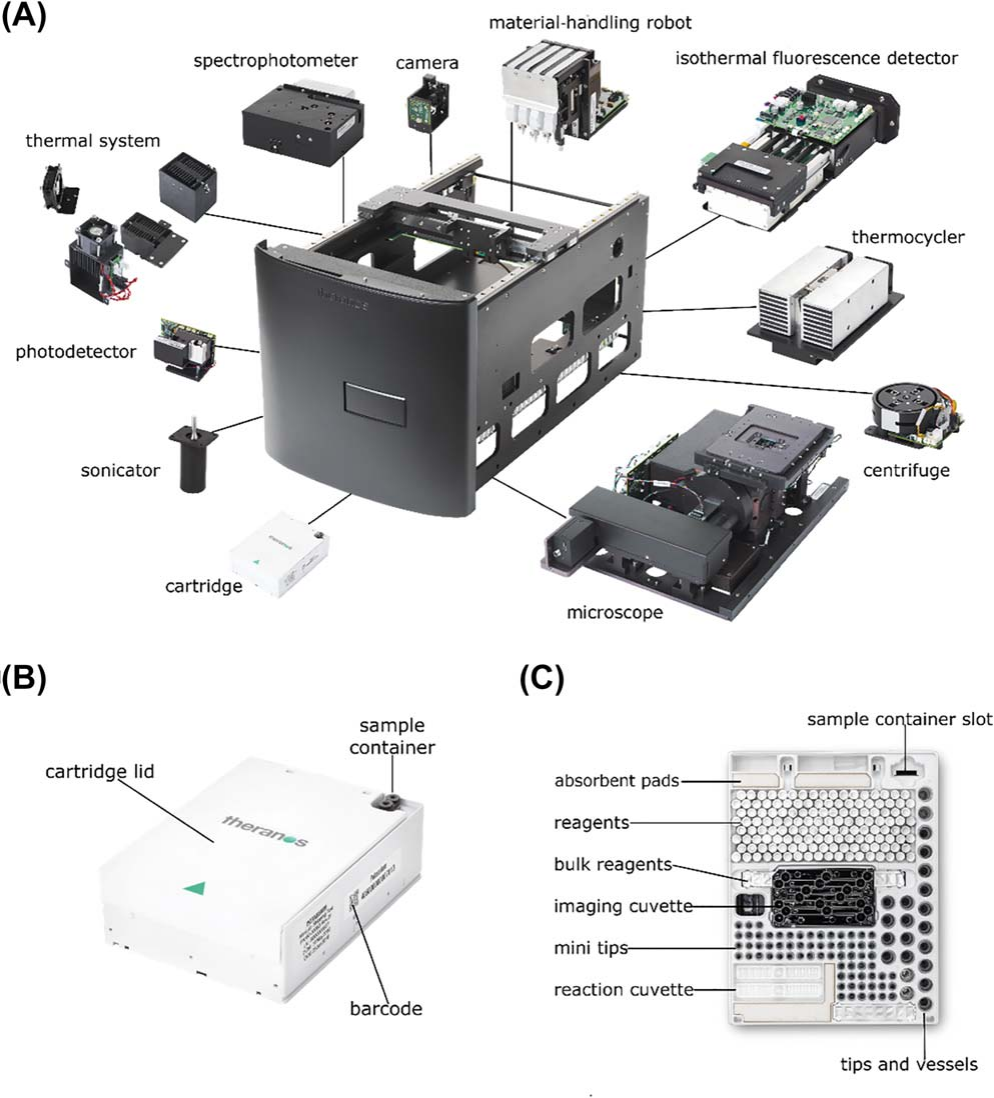
Design of the miniLab and cartridge. (A) miniLab and modules, (B) assay‐configurable cartridge in closed configuration, (C) arrangement of consumable materials within the cartridge. Images are not to scale

The miniLab is a bench top modular hardware platform with a small footprint (56 x 41 x 33 cm; up to 43 kg) that performs immunoassays, general chemistry, nucleic acid, and cellular characterization assays (Figure 2, Supporting Information Video 1). The operational environment should be 20–30°C with 20–80% relative noncondensing humidity and requires mains power and internet connectivity. After a simple software shutdown procedure that automatically locks the gantry in place, the miniLab can be moved and then reinstalled in a new location as required by the end users. The miniLab is equipped with an on‐board computer running the Windows Embedded operating system. The user runs the miniLab through an exterior touchscreen that displays a simple graphical user interface application. The on‐board computer and various modules' controller boards work collectively to carry out the assay workflow and simultaneously monitor all vital performance characteristics. Additionally, the miniLab's computer maintains connectivity to the virtual analyzer. The miniLab is designed to be used by operators with minimal prior laboratory testing experience; sample preparation is automated and integrated onboard and then the miniLab and virtual analyzer automatically generate the final results. An assay on an earlier version of the test system received a Clinical Laboratory Improvement Amendments (CLIA) waiver (K143236/CW150009).

The miniLab's material‐handling robot consists of a pipette module and a gantry module to enable pick‐and‐place and liquid transfer functions during the execution of assay‐specific protocols. The pipette module consists of three small pipettes (liquid transfer range, 2–20 μl) and one large pipette (liquid transfer range, 5–60 μl) that aspirate, dispense, and mix fluids. All four pipettes are piston‐driven air displacement pipettes. Each pipette can be independently positioned in the vertical (*Z*‐axis) direction and has independent piston actuation. The larger pipette is also capable of actuating a magnetic rod that allows for magnetic bead handling operations. Additionally, the robot manipulates the contents of the inserted cartridge, including the transfer of vessels, engagement of tips, puncturing of sealed vessels, removing plugs from vessels, and holding vessels in place during interrogation and readout in the detector modules. The pipette module is mounted on the gantry module. The gantry module translates the pipette module horizontally (*X*‐*Y* plane) relative to the rest of the miniLab with a positional resolution of 2 µm with simultaneous control of motion. The gantry module also orients and couples the pipette module to the base plate of the device, to which all other modules are mounted, and provides a means for the pipette module to access all other modules in the miniLab.

The miniLab includes an on‐board centrifuge (radius, 32.5 mm) for processing specimens with a relative centrifugal field of up to 3,000*g* and a sonicator for cell lysis. A thermal management system, composed of resistive cartridge heaters, controllable fans, and temperature sensors, monitors and controls the miniLab's internal temperature. High‐efficiency particulate air filters prevent efflux of hazardous substances. Cameras installed throughout the miniLab capture the cartridge barcode and record images of consumable materials to ensure protocols are executing as expected.

For the miniLab to have a small footprint, it incorporates four miniaturized detector modules optimized for analyzing low‐volume samples (Figure 2). Taken together, these detectors are designed to generate results for a diverse range of analyte classes. Each assay may use one or more of these detectors.

1. The thermal cycling and isothermal fluorescence detection system includes a thermocycler and a photodetector. These can be used to amplify and qualitatively detect the products of nucleic acid amplification (NAA) reactions. The thermocycler uses a thermoelectric heater with forced air cooling of the heat sink. The isothermal fluorescence detection module is composed of a compact grid of 64 independently controlled excitation/emission channels. A variable‐gain detection system is optimized for fluorescence measurements with light emitting diode (LED) excitation (600–630 nm) and a photodetector to measure epifluorescence (670–800 nm). A thermal control system of resistive heaters, heat pipes, and fans maintain the isothermal set point (34–95°C) throughout the real‐time photodetection of amplification.
2. The general‐purpose photodetector module contains optics and a high‐sensitivity optical detector to measure fluorescence and chemiluminescence. For fluorescence measurements, the LED light source excites the sample at 420–460 nm with a radiant flux of 1 pW to 1 nW. The high‐sensitivity optical detector detects fluorescence emission at 570–600 nm. For chemiluminescence measurements, the module detects light emitted with a radiant flux of 500 fW to 2 nW, and the high‐sensitivity optical detector detects the luminescence emission between 400 and 600 nm.
3. The spectrophotometer module is a crossed Czerny‐Turner spectrograph featuring a broadband pulsed‐Xenon lamp, allowing for simultaneous quantification of sample absorbance levels from 300 to 800 nm, a minimum spectral resolution of 10 nm, and better than 2.5 nm spectral accuracy.
4. The microscope module detects cells and other components in samples by epifluorescence and dark‐field microscopy with a minimum lateral resolution of 1.5 µm. The microscope includes a precision stage for scanning the sample (with 30‐μm precision in the *X*‐*Y* plane) and an independent *Z*‐axis for auto‐focusing (with 1‐μm precision). An apochromatic objective lens magnifies objects onto a high‐sensitivity image sensor. The microscope uses three laser diode light sources to excite samples, and the image sensor detects multiple spectral channels through selectable optical filters. Additionally, the module incorporates a ring light for dark‐field microscopy, allowing for the visualization of elements with differential light scattering properties.

Due to space constraints within the housing, the current miniLab chassis can only contain either the thermocycler or the microscope. Thus, the assay data presented here were collected on separate miniLab configurations.

#### 2.2.2 The assay‐configurable cartridge

The self‐contained, disposable, assay‐configurable cartridges (9.2 x 12 x 3.75 cm) are designed and preloaded to contain all consumable materials required for single or multiple assay(s) on one sample (Figure 2). Consumable materials include sealed reagents and on‐board controls, reaction and imaging cuvettes, assorted pipette tips, absorbent pads, and vessels. A lid encloses the cartridge body and contents. The sample container, which consists of two conjoined tubes that each hold up to 85 µl of sample (170 µl total), inserts into a dedicated, accessible cartridge slot. The cartridge lid uses a spring‐loaded mechanism to open automatically after insertion into the miniLab, making the contents accessible to the material‐handling robot. When the test is complete, the miniLab closes the lid and ejects the cartridge containing all used consumables and the sample for disposal. This design ensures that no fluids ever contact the hardware platform, thus limiting contamination and carryover and reducing the need for routine cleaning of the miniLab. Because the cartridge contains all necessary assay materials, the user does not need to maintain separate water, reagent, or waste tanks. Cartridges described in this study were stored at 2–8°C.

The barcode on the assay‐configurable cartridge serves two functions. First, it links to the specific protocol stored in the virtual analyzer (Figure 1). Second, it links to an assay calibration specific for each lot of cartridges. The virtual analyzer stores the assay calibration settings and uses the barcode information to identify the appropriate calibration parameters for data analysis.

#### 2.2.3 The virtual analyzer

The virtual analyzer, a central server, remotely manages and automates the workflow for all miniLabs (Figure 1). It is designed to conduct remote analysis and oversight of the test results processed in distributed locations by miniLabs. It communicates with each miniLab's computer using a secure communication channel (i.e., https protocol using Transport Layer Security 1.2) to transmit the assay protocols, receive and store raw signals, confirm the quality and integrity of the cartridge components, perform data and quality control analysis, and store results. The virtual analyzer also allows for changes in protocol and other system updates to be broadcast to existing miniLabs in the field. The virtual analyzer stores all the raw signals, results, calibration, and intermediate files in a relational database that is encrypted at rest. The data exchanged between the miniLab and the virtual analyzer are encrypted using a device‐specific certificate. Additionally, the virtual analyzer can become part of a laboratory information system. Laboratory personnel may review patient sample and QC results before authorizing the release of test results.

#### 2.2.4 System QC processes

At startup and specific intervals, the miniLab runs self‐checks of the components' functions such as monitoring temperature and successful pickup of tips or vessels. The system can be configured to use several types of controls: on‐board procedural controls, on‐board assay controls, and external assay controls. Reagent‐based procedural controls are designed to monitor hardware components, consumable materials, and individual protocol steps and can run alongside the assay‐specific portion of the protocol. Analyte‐specific assay controls may be included on the cartridge to monitor reagent loss and degradation as they are processed concurrently with each sample. Additionally, future versions of the system may use the spectrophotometer to monitor specimens for hemolysis, icterus, and lipemia during the protocol to determine assay validity for analytes sensitive to these factors (for the data presented here, these interferences were monitored externally).^32^, ^50^ External assay controls are run identically to samples ensuring that the entire analytical system is working according to specifications. Assay controls may be used to assess QC for the system. Any control that fails acceptance criteria voids the assay results. These system control processes will ultimately be applied automatically through software, preventing reporting of results at risk of compromise.

#### 2.2.5 System engineering for optimal assay performance and flexibility

The miniLab's core capability for small volume processing with the necessary flexibility to accommodate various assay types is underpinned by accurate, precise, and reliable liquid handling by the material‐handling robot, as well as small volume consumable components that are compatible with the on‐board detection systems. In selecting raw materials to manufacture the consumable components of the cartridge, we balanced the requirements of small volume handling, including surface, optical, and physical properties. Furthermore, we devised and implemented methods to mitigate evaporation of small liquid volumes, such as capping aqueous reagents with oil or wax.

Due to the wide range of analyte classes and matrices the miniLab was designed to be able to test, the material‐handling robot needed to be capable of aspirating, dispensing, and mixing diverse liquids with varying rheological properties (e.g., aqueous, high viscosity, volatile liquids, and complex fluids such as whole blood). This dictated that the pipettes on the material‐handling robot have a wide dynamic range of speed, precise control, and simultaneous pump and *Z*‐axis motion. In order to achieve the required liquid handling performance and reliability, we developed the hardware and control algorithm solutions described below.

We used custom‐engineered canted coil spring shaft seals and centerless ground piston shafts in the pipettes. We designed a plastic bearing to constrain the piston motion. We optimized the gear ratio between the piston lead screw and the motor to achieve a sufficiently high number of encoder positions per unit of piston travel. We designed custom motor control algorithms and tuned the corresponding gains to achieve good steady state error, position overshoot, speed, and trajectory tracking performance for aspirating and dispensing fluids. In order to make the pipette robust, we used a profile rail linear guide for smooth motion and added a breakout printed circuit board assembly to the motor/encoder and vent valve so that all moving conductors would be combined in a high‐flex, long‐life cable. We also had custom metal gears designed so we could weld the gears to the motor and lead screw shafts. Lastly, the custom motor control algorithms used to control pump axis motion were also used to control pipette *Z*‐axis motion and the gantry module motion in the *X*‐*Y* plane.

### 2.3 Analytical performance

To demonstrate the analytical performance of the miniLab, we performed analytical sensitivity, precision, and method comparison studies across four disparate assays that represent each of the major analyte classes and use each of the miniLab's detector modules. The functionality of the thermocycler and isothermal fluorescence detector was assessed with a Zika virus nucleic acid test; the photodetector was assessed with an anti‐HSV‐2 IgG immunoassay; the spectrophotometer was assessed with a lipid panel general chemistry assay; and the microscope was assessed with a lymphocyte subset panel hematology assay. Details of each assay methodology and workflow can be found in the Supporting Information.

#### 2.3.1 Analytical sensitivity

We developed a qualitative nucleic acid test to detect Zika virus RNA in blood samples on the miniLab platform. We evaluated the analytical sensitivity of the assay by examining the limit of detection (LoD) using Zika virus spiked into whole blood with concentrations ranging from 0 to 3,520 genomic copies/ml. A minimum of six replicates were measured for each concentration tested, followed by 20 replicates at the putative limit of detection. The lowest Zika virus RNA concentration in whole blood at which a minimum of 95% of results were positive (55 genomic copies/ml) was confirmed as the assay LoD (Table 1). The analytical sensitivity is within the range reported by other available Zika virus nucleic acid amplification tests (13.4–18,000 copies/ml as reported by individual manufacturers).^33^

**Table 1:** Analytical sensitivity of the miniLab Zika virus NAA assay^a^

Zika virus concentration, copies/ml	*N* (Positive)/*N* (Replicates)	% Positive
3,520	6/6	100
2,640	6/6	100
1,760	6/6	100
1,320	6/6	100
880	6/6	100
440	6/6	100
220	6/6	100
110	6/6	100
55	26/26	100
27.5	8/10	80
13.75	2/6	33.3
0	0/6	0

^a^The LoD was determined as the lowest Zika virus concentration that yielded a minimum of 95% positive results on the miniLab. Additional replicates measured at the putative LoD, 55 copies/ml, confirmed the assay LoD.

Abbreviations: LoD = limit of detection; NAA = nucleic acid amplification.

#### 2.3.2 Precision

We measured repeatability (within‐day and within‐miniLab), between‐day, between‐miniLab, and reproducibility (across miniLab and day) with standard deviations or percent coefficients of variation (CV) across three miniLabs over 5 days with five replicates per day at two medically relevant measurand concentrations for the anti‐HSV‐2 IgG, the lipid panel, and the lymphocyte subset panel assays (Table 2, Supporting Information Figure 1).^34^ The precision results are all within established precision goals for each assay (lipid panel)^35^, ^36^ or are comparable to those of common clinical laboratory analyzers (anti‐HSV‐2 IgG and lymphocyte subset).^37^, ^38^, ^39^

**Table 2:** Precision results for the miniLab anti‐HSV‐2 IgG, lipid panel, and lymphocyte subset panel assays at two measurand concentrations^a^

			Precision (%CV or SD)			
Measurand	Precision sample material^b^	COI or concentration	Repeat‐ability	Between‐day	Between‐miniLab	Reproducibility (95% CI)
**Herpes simplex virus type 2 (COI)**						
Anti‐HSV‐2 IgG^c^	Pool 1	0.75	7.6	0.0	1.3	7.7 (6.6, 9.3)
	Pool 2	1.06	7.3	2.1	1.6	7.7 (6.6, 9.3)
**Lipid panel (mg/dl)**						
Total cholesterol^c^	Low pool	123	1.5	0.3	0.6	1.6 (1.3, 2.1)
	High pool	332	1.7	0.5	0.9	2.0 (1.6, 2.7)
HDL‐cholesterol^c^	Low pool	40	2.0	0.0	0.7	2.1 (1.7, 2.6)
	High pool	77	2.1	0.9	1.1	2.5 (2.0, 3.4)
LDL‐cholesterol^c^	Low pool	64	3.6	0.0	1.1	3.8 (3.2, 4.7)
	High pool	180	2.2	1.0	0.0	2.4 (2.0, 2.9)
Triglycerides^c^	Low pool	81	1.9	0.5	1.7	2.6 (1.8, 4.7)
	High pool	416	3.9	2.0	0.0	4.4 (3.7, 5.4)
**Lymphocyte subsets (cells/µl)**						
Total CD3+ T cells^c^	CD4 Low	841	2.5	1.9	0.8	3.3 (2.7, 4.2)
	Normal	1274	2.4	0.0	0.8	2.5 (2.1, 3.1)
CD3+CD4+ T cells^c^	CD4 Low	153	4.6	1.9	1.8	5.3 (4.4, 6.8)
	Normal	838	2.7	0.0	0.8	2.8 (2.4, 3.4)
CD3+CD8+ T cells^c^	CD4 Low	571	3.5	1.8	1.1	4.1 (3.4, 5.1)
	Normal	380	3.2	1.3	0.0	3.5 (3.0, 4.2)
CD3‐CD19+ B cells^c^	CD4 Low	357	4.5	1.2	0.0	4.7 (4.0, 5.6)
	Normal	306	3.6	1.4	0.0	3.9 (3.3, 4.7)
CD3‐CD56+/CD16+ NK cells^c^	CD4 Low	308	5.3	1.3	2.8	6.1 (4.8, 8.4)
	Normal	193	6.2	0.7	3.0	7.0 (5.6, 9.3)
Lymphocytes^c^	CD4 Low	1533	2.7	0.8	1.2	3.1 (2.5, 4.0)
	Normal	1795	2.4	0.0	0.5	2.4 (2.1, 2.9)
**Lymphocyte subsets (% of lymphocytes)**						
Total CD3+ T cells (%)^d^	CD4 Low	54.9%	0.68	0.45	0.00	0.82 (0.68, 1.01)
	Normal	71.0%	0.65	0.34	0.34	0.81 (0.63, 1.11)
CD3+CD4+ T cells (%)^d^	CD4 Low	9.9%	0.38	0.13	0.06	0.41 (0.35, 0.49)
	Normal	46.7%	0.84	0.00	0.09	0.84 (0.72, 1.01)
CD3+CD8+ T cells (%)^d^	CD4 Low	37.2%	0.86	0.40	0.00	0.95 (0.81, 1.15)
	Normal	21.2%	0.54	0.28	0.00	0.61 (0.52, 0.75)
CD3‐CD19+ B cells (%)^d^	CD4 Low	23.3%	0.83	0.42	0.14	0.94 (0.79, 1.15)
	Normal	17.0%	0.51	0.18	0.00	0.54 (0.46, 0.65)
CD3‐CD56+/CD16+ NK cells (%)^d^	CD4 Low	20.1%	0.80	0.41	0.24	0.93 (0.77, 1.16)
	Normal	10.8%	0.62	0.00	0.32	0.70 (0.55, 0.96)

^a^Point estimates and confidence intervals are based on two‐way nested ANOVA analysis. The root mean square of the repeatability (within‐day and within‐miniLab), between‐day, and between‐miniLab precision components equals the reproducibility (across‐miniLab and across‐day).

^b^See Supporting Information for details of the precision sample materials.

^c^Precision values reported are coefficients of variation.

^d^Precision values reported are standard deviations.

Abbreviations: ANOVA = analysis of variance; COI = cutoff index; CI = confidence interval; CV = coefficient of variation; HDL = high‐density lipoprotein; HSV‐2 = herpes simplex virus type 2; IgG = immunoglobulin G; LDL = low‐density lipoprotein; SD = standard deviation.

#### 2.3.3 Method comparisons

We compared results from the miniLab anti‐HSV‐2 IgG assay^40^ for each study subject to those obtained by the reference method (Focus HerpeSelect 1 and 2 Immunoblot IgG; Table 3).^41^ From this study, the sensitivity and specificity were 94.7% (95% confidence interval [CI], 87.1–97.9%) and 100% (95% CI, 97.1–100.0%), respectively. Two samples that tested equivocal (0.8 ≤ cutoff index [COI] ≤ 1.2) on the miniLab assay were retested on a different miniLab, which confirmed the equivocal results. These equivocal results accounted for two of the four reported false negatives. These data show the miniLab anti‐HSV‐2 IgG assay performs with comparable accuracy to the reference method, with high sensitivity and specificity.

**Table 3:** Method comparison results for the miniLab anti‐HSV‐2 IgG assay^a^

	Immunoblot results (reference method), *n*			
miniLab result	Positive	Equivocal	Negative	Total
Positive	71	0	0	71
Equivocal	2	0	0	2
Negative	2	0	127	129
**Total**	75	0	127	202

^a^The comparator method was the reference standard, Focus HerpeSelect 1 and 2 Immunoblot IgG (*n* = 202).

Abbreviations: HSV‐2 = herpes simplex virus type 2; IgG = immunoglobulin G.

**Figure 3:**
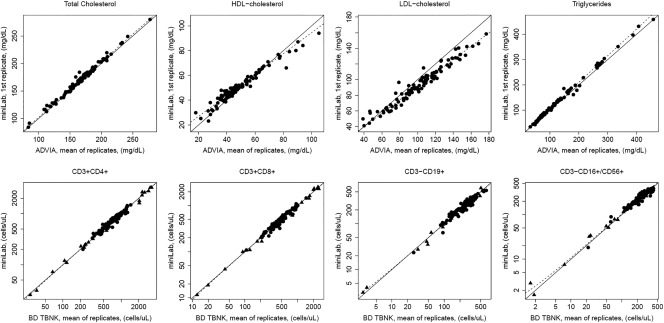
Method comparison plots showing concordance between miniLab results and comparators. Scatter plots show method comparison results for the lipid panel assay (total cholesterol, HDL‐cholesterol, LDL‐cholesterol, *n* = 103; triglycerides, *n* = 100; top row) and selected measurands from the lymphocyte subset panel (CD3 + CD4+ *n* = 116; CD3 + CD8+ *n* = 119; CD3‐CD19+ *n* = 110; CD3‐CD16+/CD56+ *n* = 95; bottom row). The dotted line represents Passing‐Bablok regression (lipid panel) or weighted Deming regression (lymphocyte subset panel). The solid line represents unity. Circles indicate native samples and triangles represent healthy samples in which leukocyte counts were either diluted or concentrated. Abbreviations: HDL = high‐density lipoprotein; LDL = low‐density lipoprotein

**Figure 4:**
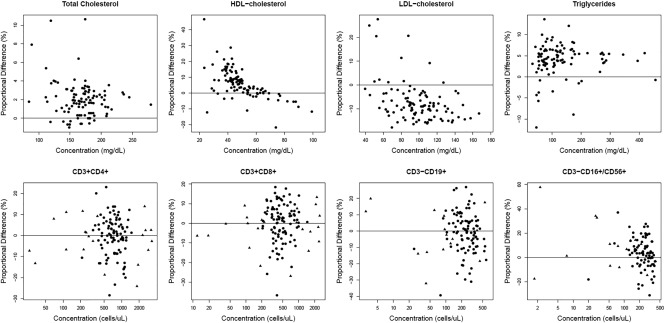
Bland‐Altman difference plots showing biases of miniLab results relative to comparator methods. The top row shows difference plots for the lipid panel measurands (total cholesterol, HDL‐cholesterol, LDL‐cholesterol *n* = 103; triglycerides *n* = 100). The bottom row shows difference plots for the lymphocyte subset panel measurands (CD3 + CD4+ *n* = 116; CD3 + CD8+ *n* = 119; CD3‐CD19+ *n* = 110; CD3‐CD16+/CD56+ *n* = 95). Circles indicate native samples and triangles represent healthy samples in which leukocyte counts were either diluted or concentrated. *X* axes represent the mean concentration of the miniLab and comparator method results. *Y* axes represent the proportional difference of the miniLab results relative to the comparator results. Abbreviations: HDL = high‐density lipoprotein; LDL = low‐density lipoprotein

For the lipid and the lymphocyte subset panels, the first result from the miniLab was plotted against the mean of duplicate results from FDA‐cleared comparator instruments for each study subject (Figure 3; Supporting Information Figure 2).^42^ The data were analyzed using either Passing‐Bablok^43^ or weighted Deming^44^ regression (Table 4). Median bias was calculated using the mean of the duplicate results (lipid panel) or the singlicate result (lymphocyte subset panel) in comparison with the mean of duplicate results from the comparator methods for each study subject (Table 3). Bland–Altman plots^45^ show the distribution of bias across each measurand concentration (Figure 4; Supporting Information Figure 3).

**Table 4:** Method comparison results for the miniLab lipid panel and lymphocyte subset panel assays^a^

Measurand	*N*	Slope (95% CI)	Intercept (95% CI)	*r*	Median absolute or proportional bias (95% CI)
Total cholesterol, mg/dl^b^	103	1.01 (0.99, 1.03)	1.29 (−2.96, 4.80)	0.99	1.90% (1.60%, 2.24%)^e^
HDL‐cholesterol, mg/dl^b^	103	0.84 (0.79, 0.89)	10.00 (7.51, 12.77)	0.98	6.18% (3.05%, 7.87%)^[Link]^, ^[Link]^
LDL‐cholesterol, mg/dl^b^	103	0.86 (0.82, 0.89)	5.56 (1.71, 9.30)	0.97	−8.94% (−10.22%, −7.91%)^e^
Triglycerides, mg/dl^b^	100	1.05 (1.04, 1.07)	−0.52 (−2.06, 0.65)	1.00	4.28% (3.77%, 5.26%)^e^
Total CD3+ T cells, cells/ul^c^	116	0.99 (0.97, 1.01)	−0.60 (−4.14, 14.64)	0.99	0.19% (−2.27%, 1.72%)^e^
CD3+/CD4+ T cells, cells/ul^c^	116	1.00 (0.97, 1.02)	−1.35 (−3.85, 10.62)	0.99	0.45% (−1.27%, 1.88%)^e^
CD3+/CD8+ T cells, cells/ul^c^	119	1.00 (0.98, 1.02)	−0.82 (−1.84, 2.71)	0.99	0.87% (−1.72%, 2.86%)^e^
CD3‐/CD19+ T cells, cells/ul^c^	110	0.98 (0.96, 1.02)	0.49 (−4.47, 0.80)	0.97	−0.55% (−3.85%, 1.45%^e^
CD3‐/CD56+/CD16+ NK cells, cells/ul^c^	95	1.04 (1.00, 1.07)	0.41 (−0.39, 3.53)	0.96	3.68% (1.31%, 6.78%)^e^
Lymphocytes, cells/ul^c^	123	1.00 (0.98, 1.02)	0.33 (−3.10, 28.96)	0.99	0.59% (−0.15%, 2.71%)^e^
Total CD3+ T cells, % lymphocytes^b^	123	0.99 (0.96, 1.03)	−0.04 (−2.72, 1.99)	0.99	−0.64 (−0.88, −0.33)^f^
CD3+/CD4+ T cells, % lymphocytes^b^	123	0.99 (0.96, 1.02)	0.05 (−1.07, 1.29)	0.99	−0.27 (−0.49, −0.04)^f^
CD3+/CD8+ T cells, % lymphocytes^b^	123	1.03 (1.01, 1.06)	−0.99 (−1.63, −0.18)	0.99	−0.11 (−0.38, 0.19)^f^
CD3‐/CD19+ B cells, % lymphocytes^b^	123	0.96 (0.93, 1.00)	0.20 (−0.29, 0.60)	0.98	−0.34 (−0.47, −0.13)^f^
CD3‐/CD56+/CD16+ NK cells, % lymphocytes^b^	123	0.97 (0.95, 1.00)	0.51 (0.05, 1.01)	0.99	0.04 (−0.13, 0.39)^f^

^a^The comparator methods were a Siemens ADVIA 1800 Clinical Chemistry Analyzer for the lipid panel and the BD Multitest^TM^ 6‐Color TBNK Reagent with Trucount Tubes for the lymphocyte subset panel.

^b^Passing‐Bablok regression.

^c^Weighted Deming regression.

^d^The bias for HDL‐cholesterol changes over the measurement interval (Figure 4). At the medical decision level of 40 mg/dl, the proportional bias is 9.3% (95% CI: 7.8%, 11.0%) and at the medical decision level of 60 mg/dl the proportional bias is 0.8% (95% CI: −0.1%, 1.8%).

^e^Median proportional bias.

^f^Median absolute bias.

Abbreviations: CI = confidence interval; HDL = high‐density lipoprotein; LDL = low‐density lipoprotein; *r* = Pearson's *r*.

Each measurand correlates well with its comparator method, and all lymphocyte subset measurands, as well as total cholesterol and triglycerides, had median biases of 4.28% or less, confirming the relative agreement of the assays (Table 4). The remaining two measurands, high‐density lipoprotein (HDL)‐cholesterol and low‐density lipoprotein (LDL)‐cholesterol, showed biases relative to the comparator method that exceed the recommended limits.^35^, ^36^

## 3 DISCUSSION

We describe the design and performance of an automated miniaturized clinical laboratory system capable of a broad test menu on a bench top instrument using small sample volumes. The miniLab's design overcomes engineering and technological challenges to meet overall size constraints, satisfy operational requirements of all elements, create and integrate hardware components, and develop control algorithms and systems that ensured seamless amalgamation of all functions. The result is a unified facile platform that incorporates multiple miniaturized detector and sample processing modules to perform laboratory tests for a variety of analyte classes on sample volumes of up to 170 µl using the same types of methods employed by central laboratory analyzers. The system is simple enough to be used by operators with minimal prior laboratory testing experience and is designed to be operated in decentralized laboratory and other near‐patient settings.

We selected tests to represent common analyte classes (molecular diagnostics, immunoassay, clinical chemistry, and hematology) and to assess each of the detector modules built into the miniLab. These tests also demonstrated the coordination and functionality of the miniLab's sample handling components (material‐handling robot, centrifuge, and magnetic bead manipulation), which together replicate many of the steps typically done by laboratory personnel in central laboratories. Most pre‐analytical and all analytical steps were self‐contained and automated within the miniLab. Our analytical sensitivity results for the Zika virus NAA assay showed that the miniLab was able to detect Zika virus at 55 copies/ml of whole blood. While this is in the same range as other tests for Zika virus RNA,^33^ it achieves this sensitivity using a sample volume of only 150 ul, meaning that the assay is able to reliably detect fewer copies of Zika virus than other tests with the same analytical sensitivity but higher sample volumes.

Precision studies for anti‐HSV‐2 IgG, lipid panel, and lymphocyte subset assays showed low imprecision for all three assays/panels and were within industry standards or similar to comparator methods. Unlike the performance characteristics of many clinical laboratory analyzers, most of the variability was captured in the repeatability component with minimal day‐to‐day or device‐to‐device variability across the three miniLab devices. Thus, the miniLabs operate consistently across devices and days.

When we compared the miniLab assays to FDA‐cleared methods, our results showed that the anti‐HSV‐2 IgG assay had high sensitivity and the lipid panel and lymphocyte subset panel had excellent correlation with their respective comparator methods, with most measurands having minimal bias. Two lipid panel analytes (HDL‐cholesterol and LDL‐cholesterol) had biases relative to the comparator method that exceed the recommended limits (Table 4).^35^, ^36^ Different methods for selectively isolating and detecting HDL‐ and LDL‐cholesterols exhibit varied biases when compared with results obtained by the reference methods, and we believe the apparent biases in this study likely reflect the inherent biases of both the comparator and miniLab methods.^35^, ^46^, ^47^, ^48^

One benefit of the miniaturized clinical laboratory system is its ability to conduct newly developed tests. The miniLab hardware can accept any new assay‐configurable cartridge and download the protocol to manipulate the contents of the cartridge from the virtual analyzer. As new tests become available for a given miniLab configuration, the device itself does not require any modifications. Another advantage is that the system is designed to be able to multiplex tests that comprise frequent ordering patterns, including those from different analyte classes, on each sample. The number of tests the system may ultimately be able to multiplex on a single sample depends on the sample volume and type required for each test, number of reagents and space available on the cartridge, and overlap of requirements between test types. We envision all assays functioning with blood specimens either in the absence of anticoagulants (serum) or the presence of a limited set of common assay‐compatible anticoagulants (i.e., lithium heparin, dipotassium ethylenediaminetetraacetic acid, and sodium citrate). Limiting the number of anticoagulants may increase the potential to multiplex tests across analyte classes and could reduce the number of separate specimens required for larger test order sets. In addition, the system is compatible with other specimen types such as urine. Finally, if desired, laboratory personnel may remotely oversee and interpret control and test results through the virtual analyzer, thus allowing for a similar review as is typically available in a clinical laboratory setting.

The design of the system presents some limitations. As with certain point of care analyzers, the miniLab can test only one cartridge at a time, which limits the throughput of a single device. We plan to shorten the assay run times to increase the miniLab's throughput and utility. The data presented here show only one assay or panel per cartridge, though the system is designed to be capable of multiplexing disparate assays or panels together using a single cartridge and associated protocol. Finally, the current version of the platform cannot contain all the modules in the same chassis. Future versions of the platform will contain the full functionality of all modules within a single device.

## 4 CONCLUSIONS

The miniLab has a unique potential as a clinical laboratory testing platform, and the system is designed to fill several unmet opportunities in the field of diagnostic testing. The system requires smaller specimen volumes than many other analyzers. This makes frequent testing more feasible for populations for whom required blood volume may limit laboratory testing (e.g., the young, old, and select at‐risk populations). The miniLab's compact size, coupled with the virtual analyzer's connectivity and the versatile assay‐configurable cartridges, has the potential to provide high‐quality testing of diverse analytes in decentralized laboratory and other near‐patient locations, which could expand access to clinical services and expedite diagnoses and therapies. By augmenting access to clinical laboratory testing options, the miniaturized clinical laboratory system has the potential to complement the arsenal of technologies available to the clinical laboratory community.

## 5 MATERIALS AND METHODS

Extended methods and Supporting Information Figures, Tables, and Video are available online.

To demonstrate the analytical capabilities of each detector module, we performed the following illustrative assays on the miniLab using automated protocols: Zika virus NAA test (for the thermal cycling and isothermal fluorescence detection system), anti‐HSV‐2 IgG (for the photodetector), lipid panel (total cholesterol, triglycerides, HDL‐cholesterol, LDL‐cholesterol concentrations; for the spectrophotometer), and lymphocyte subset panel (concentrations and percentages of total CD3+ T cells, CD3 + CD4+ [CD4 + T cells], CD3 + CD8+ [CD8 + T cells], CD3‐CD19+ [B cells], CD3‐CD16+/CD56+ [NK cells], and CD45 + SSC^Low^ [lymphocyte count]; for the microscope). All miniLab assays were conducted by loading up to 160 µl of sample into an assay‐configurable cartridge and inserting it into the miniLab. Basic assay specifications (see Supporting Information Table 1) and extended details of each assay methodology are provided in the Supporting Information.

We assessed the analytical sensitivity of the qualitative Zika virus NAA assay for detecting the virus in venous whole blood (Supporting Information Figure 4). For real‐time monitoring of the isothermal amplification, the inflection cut‐off time was taken as the time at which 92.6% sensitivity (positive sample detection rate) and 95.0% specificity (rate of correctly identified negative samples) were achieved via receiver operating characteristic analysis. The LoD study design followed the March 9, 2016, FDA draft interactive emergency use authorization review template for molecular assays (Zika virus‐specific). The preliminary LoD concentration was determined by testing contrived venous whole blood samples of decreasing Zika virus concentrations in replicates of six or more until the assay no longer detected the virus at the tested concentration. The LoD was confirmed by identifying the lowest concentration exhibiting at least a 95% Zika virus detection rate after testing an additional 20 replicates.

For the anti‐HSV‐2 IgG, lipid panel, and lymphocyte subset panel tests, we measured precision by testing two sample pools at low and high measurand concentrations over 5 days, with five replicates each day on each of three miniLabs, following the multidevice precision guidelines in the Clinical Laboratory Standards Institute (CLSI) EP05‐A3 (see Supporting Information Figures 1 and 5–7).^34^

We performed method comparison studies for the anti‐HSV‐2 IgG, lipid panel, and lymphocyte subset panel assays by testing a minimum of 100 samples on the miniLab and an FDA‐cleared comparator or reference method.^41^, ^42^ For anti‐HSV‐2 IgG, we tested intended‐use specimens in one replicate each on the miniLab and the reference method (Focus HerpeSelect 1 and 2 Immunoblot IgG; Supporting Information Figure 5). We analyzed the data by assessing the sensitivity and specificity of the miniLab results with respect to the reference method.

We tested fresh and archived plasma clinical specimens in duplicate on the miniLab and the comparator method (Siemens Healthcare ADVIA 1800 Chemistry System; Siemens Healthcare Diagnostics, Inc., Tarrytown, NY) for the lipid panel method comparison study (Supporting Information Figure 6). We tested fresh venous whole blood and contrived samples in singlicate on the miniLab and in duplicate on the comparator method, Becton Dickinson (BD) Biosciences Multitest 6‐Color TBNK (BD Biosciences, San Jose, CA; see Supporting Information for additional details), for the lymphocyte subset method comparison study (Supporting Information Figure 7).

We purchased all anti‐HSV‐2 IgG specimens (202 samples) and some lipid panel specimens (21 samples) used for method comparison studies as de‐identified clinical specimens from commercial vendors (see Supporting Information). We collected blood specimens for all other studies from 224 healthy adult donors by venipuncture using BD Vacutainers (Becton, Dickinson, and Company, Franklin Lakes, NJ). Veritas Institutional Review Board, Inc. approved the study protocols. All subjects gave informed consent, and consent was waived for commercially purchased specimens. Theranos manufactured all miniLabs, reagents, cartridges, and consumable materials for each assay in Newark, CA.

### 5.1 Statistical analysis

Prior to analysis, invalid miniLab results were determined by applying the following exclusion criteria: tests which failed to collect all assay data, flagged integrity checks (on‐board controls out of bounds, intra‐cartridge assay replicate disagreement, data integrity checks, or sample integrity checks), and traceable human error. Results from comparator methods that exceeded the analytical measuring range as reported in the package inserts were also excluded, as well as any incomplete data sets. In some cases of excluded tests where sample volume and time permitted, samples were retested on a new cartridge.

Statistical analysis was performed with R statistical software (The R Foundation for Statistical Computing, Vienna, Austria). The precision analysis was performed with two‐way nested analysis of variance with random effects (“day” nested within “device”) to determine the components of the variance for each assay. We used Grubbs' test at the 99% level to exclude up to one outlier per miniLab per measurand and per sample pool, as recommended in the CLSI EP05‐A3 guideline.^34^ We estimated the repeatability (within‐day and within‐miniLab), between‐day variation, between‐miniLab variation, and reproducibility (across‐day and across‐miniLab; across three miniLabs) and their 95% CIs for these estimates. The root mean square of the repeatability, between‐day, and between‐miniLab precision components equals the reproducibility (across‐miniLab and across‐day). We used the Satterthwaite approximation to calculate the 95% CIs on the precision terms.^34^

The sensitivity, specificity, and their CIs (direct score calculation) were calculated as described in CLSI EP12‐A.^41^

Quantitative method comparison of the first replicate (lipid panel) or singlicate (lymphocyte subset) result from the miniLab and mean of duplicate results from the comparator methods^41^ was performed by Passing‐Bablok^43^ regression (for lipid panel and lymphocyte subset percentages) or weighted Deming^44^ regression (for lymphocyte subset concentrations). Ninety‐five percent CIs for the slope and intercept for each regression were calculated by the bootstrap method. Weighted Deming regression was used for analytes with wide dynamic ranges that were operating in the constant CV region, as recommended by CLSI guideline EP09‐A3.^42^ We estimated linear correlation by calculating the Pearson correlation coefficient (*r*) for each measurand.

For bias calculations and plots, mean of duplicate (lipid panel) or singlicate (lymphocyte subset panel) miniLab results were compared to the mean of duplicate results for the comparator method for each study subject:

Absolute bias = miniLab−comparator method

$$Relative\;bias\;=\frac{miniLab-comparator\;method}{(miniLab+comparator\;method)/2}$$

Median absolute bias or median proportional bias was calculated and the approximate 95% CI was obtained by an application of the binomial distribution.^49^

### 5.2 Data availability

The data and analysis scripts presented in this paper are available at https://osf.io/ur7kw/

## CONFLICT OF INTEREST

All authors of this paper, as either current or former Theranos employees, may have received or may in the future receive equity compensation from the Company. S.G.A., K.E., E.A.H., T.M.K., A.N., M.B.N., J.O., C.H.P., P.J.P., J.R., C.S., S.S., B.S.‐P., and D.L.Y. are inventors on patents or patent applications related to the material presented here, all of which are assigned to Theranos.

## AUTHOR CONTRIBUTIONS

S.G.A., P.B., S. Chow, K.D.H., E.A.H., J.O., C.H.P., J.R., N.V.S., C.S., L.T., T.C.W., P.Z. designed and/or optimized functional components of the miniLab device. S.G.A., K.D.H., E.A.H., J.O., C.H.P., J.R., C.S., B.S.‐P., A.T. designed and/or optimized consumable materials contained in the miniLab cartridge. S. Chandrasekaran, E.G., X.G., K.J., R.S. wrote software for the miniLab and/or virtual analyzer. S. Chandrasekaran, X.G., A.J., K.J., J.O. wrote software to generate miniLab protocols. K.Q., T.C.W. designed and implemented the embedded control software for custom electronics. S.G.A., S. Chow, X.G., K.D.H., J.O., J.R., J.F.S., A.T., T.C.W., P.Z. optimized hardware integration and control processes for miniLab. D.P.B., X.G., K.D.H., K.J., A.N., J.O., C.H.P., J.F.S., A.T., D.W., A.Y. wrote and optimized protocol instructions for the miniLab. S.G.A., D.P.B., Y.C., U.D., K.E., S.F.G., K.D.H., E.A.H., R.H., L.H., A.N., M.B.N., J.O., C.H.P., A.R.R., J.F.S., N.V.S., C.S., S.S., L.T., A.T., D.W. designed and/or optimized the assay methods. T.M.K. specified requirements for anti‐HSV‐2 IgG assay calibration. Y.C., U.D., K.E., K.D.H., R.H., L.H., Y.L.L., M.B.N., J.O., C.H.P., P.L.R., A.R.R., J.F.S., N.V.S., C.S., B.S.‐P., L.T., A.T., D.W. developed and/or optimized assay reagents. D.P.B., Y.C., S. Chow, K.E., S.F.G., X.G., K.D.H., R.H., L.H., K.J., A.N., M.B.N., J.O., P.J.P., A.R.R., J.F.S., N.V.S., C.S., S.S., B.S.‐P., D.L.Y., designed experiments and/or research plans. J.A.B., D.P.B., Y.C., K.E., S.F.G., K.D.H., R.H., L.H., A.N.K., L.S.L., Y.L.L., A.N., J.O., A.R.R., J.F.S., C.S., L.T., A.T., D.W. performed experiments. J.A.B., D.P.B., Y.C., K.E., E.G., S.F.G., X.G., K.D.H., L.H., A.J., K.J., A.N.K., M.B.N., J.O., A.R.R., J.F.S., N.V.S., C.S., B.S.‐P., D.W. analyzed and visualized data. S.G.A., J.A.B., D.P.B., Y.C., S. Chow, K.E., S.F.G., X.G., R.H., K.J., A.N.K., Y.L.L., M.B.N., J.O., A.R.R., J.F.S., N.V.S., C.S., S.S., B.S.‐P., A.T., D.W., D.L.Y. discussed and interpreted results. C.H.P., P.J.P., N.V.S., C.S., S.S., D.L.Y. supervised projects. E.A.H., C.H.P., D.L.Y. conceived the overall system design. K.E., M.B.N., C.R.R. wrote the manuscript. All authors critically reviewed and/or revised the manuscript. All authors have read and approved the final version of the manuscript.

## Supporting information

Additional Supporting Information may be found online in the supporting information tab for this article.

Supporting Information Video

Supporting Information
