# Supplementary material for: Engineering of a miniaturized, robotic clinical laboratory
Source: Bioeng Transl Med. 2018 Jan 19;3(1):58–70. doi: 10.1002/btm2.10084 (PMC5773944; doi:10.1002/btm2.10084)
Supplement: Supplementary file 2 — Supporting Information [file BTM2-3-58-s002.docx]

**Engineering of a miniaturized, robotic clinical laboratory**

**Supporting Information**

**Table of Contents**

| **Section** | **Page Number** |
| --- | --- |
| 1 MATERIALS AND METHODS | 2 |
| 1.1 Workflow | 2 |
| 1.2 Exclusion criteria | 2 |
| 1.3 miniLab Zika virus nucleic acid amplification test | 2 |
| 1.4 Zika virus quantitation | 3 |
| 1.5 miniLab herpes simplex virus type 2 immunoglobulin G assay | 4 |
| 1.6 miniLab lipid panel | 6 |
| 1.7 miniLab lymphocyte subset panel | 8 |
| SUPPORTING INFORMATION LITERATURE CITED | 11 |
| SUPPORTING INFORMATION TABLE 1 | 12 |
| SUPPORTING INFORMATION FIGURE 1 | 13 |
| SUPPORTING INFORMATION FIGURE 2 | 14 |
| SUPPORTING INFORMATION FIGURE 3 | 15 |
| SUPPORTING INFORMATION FIGURE 4 | 16 |
| SUPPORTING INFORMATION FIGURE 5 | 17 |
| SUPPORTING INFORMATION FIGURE 6 | 18 |
| SUPPORTING INFORMATION FIGURE 7 | 19 |
| SUPPORTING INFORMATION VIDEO 1 | 20 |

**1 MATERIALS AND METHODS**

- 1. **Workflow**

For this study on the miniLab, two 80 μL aliquots of the relevant sample were transferred into the dual-chamber sample container (160 μL total) which was then inserted into the cartridge, and then the cartridge was inserted into the miniLab. For anti-HSV-2 cartridges, a 30 μL sample was transferred directly into a small empty vessel in the cartridge and then the cartridge was inserted into the miniLab. Following protocol initiation no further intervention was required by the operator.

**1.2 Exclusion criteria**

Prior to analysis, invalid miniLab results were determined by applying the following exclusion criteria: tests which failed to collect all assay data, flagged integrity checks (on-board controls out of bounds, intra-cartridge assay replicate disagreement, data integrity checks, or sample integrity checks), and traceable human error. Results from comparator methods that exceeded the analytical measuring range as reported in the package inserts were also excluded, as well as any incomplete datasets. In some cases of excluded tests where sample volume and time permitted, samples were retested on a new cartridge.

**1.3 miniLab Zika virus nucleic acid amplification test**

The Zika virus nucleic acid amplification test workflow to determine analytical sensitivity is summarized in Supporting Information Figure 4.

Venous whole blood spiked with Zika virus (80 µL) was loaded into each of the 2 sample container tubes (160 µL total). The miniLab added sample processing control and hemolyzing agents to 150 µL of whole blood. Samples were spun in a centrifuge and 75 μL of plasma was subjected to lysis. RNA was extracted through capture onto MagBinding Beads (Zymo Research, Irvine, CA), washed, and then eluted into water. Extracted RNA and proprietary preliminary amplification master mix were combined in a reaction vessel, and the vessel was transferred to the thermal-cycling module to perform reverse transcription polymerase chain reaction (RT-PCR preliminary amplification). Three microliters of the amplified products were added to vessels containing the isothermal reaction master mix for a final volume of 25 µL. The isothermal amplification/detection reaction was performed at 56°C. The primer pairs contained pair-wise complementary 5’ ends that resulted in amplicons containing 5’ overhangs. These overhangs facilitated the generation of concatemers, which were detectable with intercalating fluorescence dye. The assay executed in 140 minutes.

Samples for the analytical sensitivity study were prepared by spiking K_2_ ethylenediaminetetraacetic acid (K_2_-EDTA) anticoagulated whole blood with Zika virus (strain PRVABC59 obtained from the Centers for Disease Control and Prevention, Atlanta, GA) at the indicated test concentrations. Seven miniLabs were used to perform the study. The result from 1 run met the exclusion criteria (flagged integrity check), and this sample was retested.

**1.4 Zika virus quantitation**

A synthetic RNA sequence was designed based on a consensus Zika RNA sequence (includes portions of Genbank sequence #KU991811), synthesized (Integrated DNA Technologies, Inc., Redwood City, CA), and quantified using the Qubit RNA HS Assay Kit on the Qubit Fluorometer (Thermo Fisher, Waltham, MA) per the manufacturer’s protocol. The concentration units were converted to copies/µL using the sequence’s molecular weight. This synthetic RNA was then used as the standard for quantitative RT-PCR (RT-qPCR).

RNA from 3 aliquots of Zika PRVABC59 Strain at 1x10^6^ PFU/mL was purified using the Qiagen QIAcube® Robotic workstation with Qiagen spin column kits (Qiagen, Germantown, MD). The purified RNA from each aliquot was diluted 10, 30, and 100-fold and quantified by RT-qPCR using ABI Power SYBR Green Ct-to-one-step RNA kit. Zika virus stock concentration was back-calculated, and certain concentrations were spiked into the whole blood for the Zika assay analytical sensitivity study on the miniLab.

**1.5 miniLab** **anti-herpes simplex virus type 2 immunoglobulin G assay**

The workflows for the anti-herpes simplex virus type 2 (HSV-2) immunoglobulin G (IgG) precision and method comparison studies are summarized in Supporting Information Figure 5.

To perform the miniLab anti-HSV-2 IgG assay, each HSV-2 cartridge was loaded with 30 µL of serum. The miniLab aspirated 4 µL of the sample to perform the test. The anti-HSV-2 IgG test was a 3-step immunoassay with an HSV-2 glycoprotein G (gG2) recombinant antigen^1^ coated magnetic beads, an anti-human IgG (Novus Biologicals Inc., Littleton, CO) detection reagent conjugated to alkaline phosphatase (AP), and a chemiluminescent substrate. During the first incubation step, anti-HSV-2 antibodies in the sample bound to the gG2 recombinant antigen on the coated magnetic beads. Following the first incubation step, unbound materials were removed with a wash cycle. Then, the anti-human IgG-AP conjugate was added, which bound to the anti-HSV-2 IgG antibodies captured by the antigen. After the second incubation, unbound materials were removed with a wash cycle, and the chemiluminescent substrate was added to the capture-analyte-detection complex to initiate the chemiluminescence reaction. The photodetector module measured the chemiluminescence. The assay executed in 80 minutes.

Each miniLab was calibrated using 4 calibrators across the analytical range to establish the miniLab cutoff relative light units (RLU) value. The test result was normalized to the light intensity of the cutoff value on each miniLab to determine the specimen cutoff index (COI) as shown below:

$$Specimen COI=\frac{Specimen RLU}{device-specific cutoff RLU}$$

Test results were defined as positive (a COI > 1.2), negative (a COI < 0.8), or equivocal (a 0.8 ≤ COI ≤ 1.2).

The precision study samples were 2 pools close to the assay cut-off (pool 1 COI, 0.75; pool 2 COI, 1.06), created by blending anti-HSV-2 IgG positive serum (Plasma Services Group, Huntingdon Valley, PA) and anti-HSV-2 IgG negative sera (BioreclamationIVT, New Cassel, NY). The precision results from 11 runs met the exclusion criteria (3 protocol non-completions, 2 flagged integrity checks, 6 traceable human errors); all of these samples were retested. The method comparison study samples were 202 remnant intended-use specimens (venous serum samples from patients with lab orders for HSV-1/2; Cureline, South San Francisco, CA and Discovery Life Sciences, Los Osos, CA). The reference standard was the Focus Diagnostics HerpeSelect 1 and 2 Immunoblot IgG test. Seven miniLabs were used to perform the method comparison study. All samples that tested as equivocal in the method comparison study were retested to confirm the result. The method comparison results from 21 runs met the exclusion criteria (10 protocol non-completions, 11 flagged integrity checks); all of these samples were retested.

**1.6 miniLab lipid panel**

The workflow of the lipid panel precision and method comparison studies are summarized in Supporting Information Figure 6.

Venous lithium heparin plasma (80 µL) was loaded into each of 2 sample container tubes (160 µL total). The miniLab aspirated 8 µL of plasma to perform the assay. The assay used a series of coupled enzyme peroxidase-based Trinder reactions, for which the readout quantifies a quinonimine dye measured at 500 nm (total cholesterol) or 545 nm (high-density lipoprotein [HDL]-cholesterol, low-density lipoprotein [LDL]-cholesterol, and triglycerides). Total cholesterol measurements used a coupled, cholesterol esterase-cholesterol oxidase-peroxidase reaction.^2^ Concentrations of HDL-cholesterol were measured directly in a 2-step process. In the first step, non-HDL cholesterol particles were selectively blocked by dextran sulfate, α-cyclodextrin sulfate, and magnesium chloride; in the second, HDL-cholesterol particles were solubilized by surfactant and measured using a coupled PEGylated cholesterol esterase-cholesterol-oxidase-peroxidase reaction.^3^ Concentrations of LDL-cholesterol were also measured directly in a 2-step process. In the first step, non-LDL-cholesterol particles were removed by adding selective detergents and enzymatic depletion using a coupled cholesterol esterase-cholesterol oxidase-catalase reaction.^4,5^ In the second step, azide, additional detergents, and peroxidase were added to solubilize and detect LDL-cholesterol selectively. Triglycerides measurements used a coupled lipase-glycerol kinase-glycerol-3-phosphate oxidase-peroxidase reaction.^6,7^ Each miniLab was calibrated using 4 levels of calibrators with concentrations assigned by measuring the average from 5 different commercial analyzers. Calibration determined the linear relationship between absorbance and analyte concentration. Absorbance results were automatically processed by the virtual analyzer to calculate sample concentrations. The assay executed in 45 minutes.

Samples for lipid panel precision were formulated by blending and modifying venous plasma with lithium heparin pools with human cholesterol and triglycerides fractions (Creative Laboratory Products, Indianapolis, IN). One sample was clinically low for all lipids (total cholesterol, 120 mg/dL; HDL-cholesterol, 40 mg/dL; LDL-cholesterol, 71 mg/dL; triglycerides, 78 mg/dL), and the second sample was clinically high for all lipids (total cholesterol, 320 mg/dL; HDL-cholesterol, 84 mg/dL; LDL cholesterol, 203 mg/dL; triglycerides, 405 mg/dL). Three of the runs in the precision studies met the exclusion criteria (2 protocol non-completions, 1 flagged integrity check); all of these samples were retested. During analysis, 1 outlier (total cholesterol, low pool and high pool; HDL-cholesterol, low pool; LDL-cholesterol, high pool; triglycerides, low pool) or 2 outliers (HDL-cholesterol, high pool) were identified by Grubbs’ test at the 99% level and excluded from analysis. The lipid panel method comparison studies were performed by duplicate testing of 107 samples on the miniLab and the Siemens ADVIA 1800 Chemistry system (total cholesterol, direct HDL-cholesterol, direct LDL-cholesterol, and triglycerides assays). Nine miniLabs were used to execute the method comparison study. All samples were venous plasma with lithium heparin. Of the 107 samples, 86 were collected from apparently healthy donors, who were expected to have clinically normal lipid concentrations. The remaining 21 samples had clinically abnormal lipid concentrations and were acquired from ProMedDx (Norton, MA) and iSpecimen (Lexington, MA). Thirteen of the runs in the method comparison study met the exclusion criteria (1 protocol non-completion, 10 flagged integrity checks; 2 incomplete data sets); 7 runs were repeated, and the results from the remaining 6 runs (data from 4 subjects) were excluded from analysis. Results that fell outside the comparator method’s reported analytical range were also excluded (3 results for triglycerides).

To determine the bias at the medical decision levels for HDL-cholesterol (Table 4, footnote “e”), Passing-Bablok regression was performed comparing the mean of duplicate results from the miniLab and the mean of duplicate results from the comparator method. The proportional bias (*y – x*)*/((x+y)/2)* at each medical decision level (*x*) was determined from the linear regression equation (*y = a + bx,* where *a* is the intercept and *b* is the slope). Ninety-five percent confidence intervals for the bias were calculated by the bootstrap method.

**1.7 miniLab lymphocyte subset panel**

The workflow for the lymphocyte subset precision and method comparison studies are summarized in Supporting Information Figure 7.

K_2_-EDTA venous whole blood (80 µL) was loaded into each of the 2 sample container tubes (160 µL total), and the miniLab aspirated 31 µL of the sample to perform the lymphocyte subset panel. Polystyrene beads (SureCount Particle Count Standards, 10-μm diameter, Polysciences, Warrington, PA), were added to each reaction to serve as a fiducial concentration. Lymphocyte subset measurements were determined with direct immunofluorescence for CD3 (clone UCHT1, BioLegend, San Diego, CA, conjugated to Pacific Blue), CD4 (clone SK3, BioLegend, conjugated to R-phycoerythrin [PE]), CD8 (clone Hit8a, BioLegend, PE-Cy5), CD14 (clone 60bca, ATCC, Pacific Orange), CD16 (clone 3G8, BioLegend, PE), CD19 (clone CAT-13.1E10, DSMZ: German Collection of Microorganisms and Cell Cultures, GmbH, PE-Cy5, Braunschweig, Germany), CD45 (clone 9.4, ATCC, allophycocyanin), CD56 (clone NCAM16.2, BD Biosciences, PE, San Jose, CA), and a nuclear stain (DRAQ5, eBioscience, San Diego, CA) in 2 separate reactions (Reaction A: CD45/CD3/CD14/CD4/CD8, Reaction B: CD45/CD3/CD14/CD19/CD16/CD56). Bead reagent concentration was determined by particle counting on a Z2 Coulter Counter (Beckman Coulter, Brea, CA).

Erythrocytes were lysed with a detergent-based lysing agent, and leukocytes were fixed with formaldehyde. Samples were loaded into a slide and imaged with fluorescence and dark‑field microscopy. The assay executed in 109 minutes. Images were segmented, resulting in cell and bead regions of interest (ROIs) for each field of view. Intensity values for each cell were extracted from each ROI and aggregated into flow cytometry standard files. Lymphocyte subset results were analyzed using FlowJo software v7.6.5 (FlowJo LLC, Ashland, OR). For each sample, the concentration of lymphocytes was calculated as:

$${Lymph conc}_{A or B}= \frac{Lymphocyte ROIs}{Bead ROIs}X\frac{Bead volume}{Blood volume}X bead concentration$$

The final lymphocyte concentration was taken as the average from reaction A and reaction B.

For lymphocyte subsets, each subset percentage was taken in reference to the number of total lymphocytes:

$$Subset (\%)= \frac{Cell subset events}{Lymphocyte events}\times100$$

The total CD3% was then averaged for reaction A and reaction B. For each subset, the absolute concentration was calculated as:

$$Subset conc.=\frac{Subset (\%) \times Lymphocyte concentration}{100}$$

Samples for the precision study were commercially available, stabilized, normal and low-CD4 whole blood samples from R&D Systems (StatusFlow and StatusFlow^LO^, Minneapolis, MN). Seven runs in the precision study met the exclusion criteria (4 protocol non-completions, 3 flagged integrity checks); 4 of these samples were retested. For the lymphocyte subset method comparison, we compared 128 K_2_-EDTA whole blood samples (97 native venous and 31 adjusted to abnormal concentrations). Each sample was measured once on the miniLab and twice on the BD FACSCanto II (Multitest 6-color TBNK Reagent with Trucount tubes, BD Biosciences, San Jose, CA). Seven miniLabs were used to perform the method comparison study. Seven runs in the method comparison study met the exclusion criteria (3 protocol non-completions, 4 flagged integrity checks); 2 of these samples were retested, and the remaining 5 subjects were excluded from analysis. To assess performance outside the normal range, samples with leukocyte concentrations above and below the reference range (4 to 11 x 10^3^ leukocytes/μL) were generated by spinning whole blood in a centrifuge (at 1200 *g* for 5 minutes) and adjusting the amount of buffy coat to alter the effective leukocyte concentration while maintaining a hematocrit similar to the original sample. Results that fell outside the comparator method’s reported analytical measuring range were also excluded (7 results for Total CD3+ cells/μL; 7 results for CD3+CD4+ cells/μL; 4 results for CD3+CD8+ cells/μL; 13 results for CD3-CD19+ B cells/μL, and 28 results for CD3-CD16+/CD56+ cells/μL).

**SUPPORTING INFORMATION LITERATURE CITED**

1. Jun W, Hu R, Hyland L, et al. Expression and characterization of the soluble form of recombinant mature HSV-2 glycoprotein G for use in anti-HSV-2 IgG serodiagnostic immunoassay. *J Virol Methods*. Published October 28, 2017. doi: 10.1016/j.jviromet.2017.10.021.

2. Allain CC, Poon LS, Chan CS, Richmond W, Fu PC. Enzymatic determination of total serum cholesterol. *Clin Chem.* 1974;20(4):470-5.

3. Warnick GR, Benderson J, Albers JJ. Dextran sulfate-Mg2+ precipitation procedure for quantitation of high-density-lipoprotein cholesterol. *Clin Chem.* 1982;28(6):1379-88.

4. Nauck M, Warnick GR, Rifai N. Methods for measurement of LDL-cholesterol: a critical assessment of direct measurement by homogeneous assays versus calculation. *Clin Chem.* 2002;48(2):236-54.

5. Okada M, Matsui H, Ito Y, Fujiwara A, Inano K. Low-density lipoprotein cholesterol can be chemically measured: a new superior method. *J Lab Clin Med.* 1998;132(3):195-201.

6. Fossati P, Prencipe L. Serum triglycerides determined colorimetrically with an enzyme that produces hydrogen peroxide. *Clin Chem.* 1982;28(10):2077-80.

7. McGowan MW, Artiss JD, Strandbergh DR, Zak B. A peroxidase-coupled method for the colorimetric determination of serum triglycerides. *Clin Chem.* 1983;29(3):538-42.

**SUPPORTING INFORMATION TABLES**

SUPPORTING INFORMATION TABLE 1 Properties of the Zika virus nucleic acid test, Anti-HSV-2 IgG, lipid panel, and lymphocyte subset panel assays on the miniLab.

| **Assay** | **Sample Type** | **Sample Volume Loaded (µL)** | **Sample Volume Used (µL)*^a^*** | **Detector Module** | **Assay Run Time (min)** |
| --- | --- | --- | --- | --- | --- |
| Zika virus nucleic acid | K_2_-EDTA whole blood*^b^* | 160 | 150  (75 plasma) | Thermocycler and isothermal  fluorescence detector | 140 |
| Anti-HSV-2 IgG | Serum | 30 | 4 | Photodetector | 80 |
| Lipid panel | Lithium heparin plasma | 160 | 8 | Spectrophotometer | 45 |
| Lymphocyte subset panel | K_2_-EDTA whole blood | 160 | 31 | Microscope | 109 |
|  |  |  |  |  |  |
| *^a^*Volume used during the miniLab protocol to perform the assay.  *^b^*Whole blood centrifuged and separated to plasma on board.  Abbreviations: HSV-2, herpes simplex virus type 2; IgG, immunoglobulin G; EDTA, ethylenediaminetetraacetic acid. | | | | | |

SUPPORTING INFORMATION FIGURE 1 Comb diagram representing precision study design.*

*Each concentration of precision control material was tested on 3 miniLabs for 5 days with 5 replicates per day.

SUPPORTING INFORMATION FIGURE 2 Additional method comparison plots showing concordance between miniLab results and comparators.*


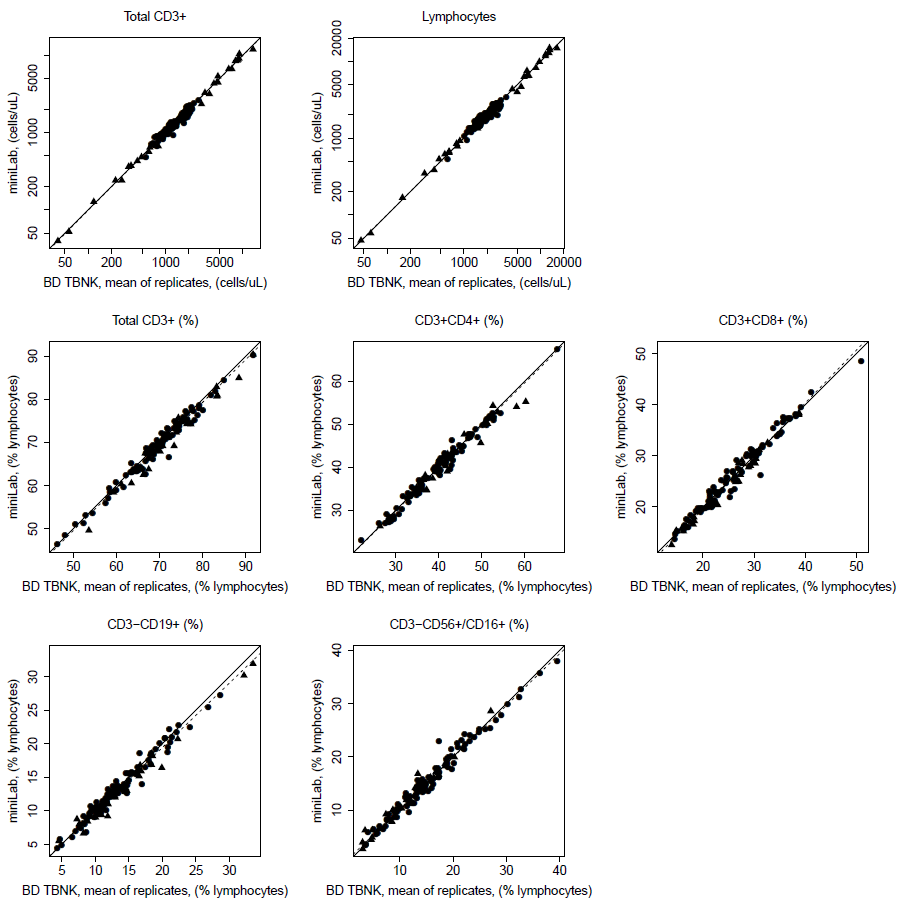


*Scatter plots show method comparison results for selected measurands from the lymphocyte subset panel (Total CD3+, n = 116; remaining measurands, n = 123). The dotted line represents weighted Deming regressions for the lymphocyte subset panel of Total CD3+, Lymphocytes, and Passing-Bablok regressions for Total CD3+ (%), CD3+CD4+ (%), CD3+CD8+ (%),CD3-CD19+ (%), CD3-CD56+/CD16+ (%). The solid line represents unity. Circles indicate native samples and triangles represent healthy samples in which leukocyte counts were either diluted or concentrated.

SUPPORTING INFORMATION FIGURE 3 Additional Bland-Altman difference plots showing biases of miniLab lymphocyte subset panel results relative to comparator method.*


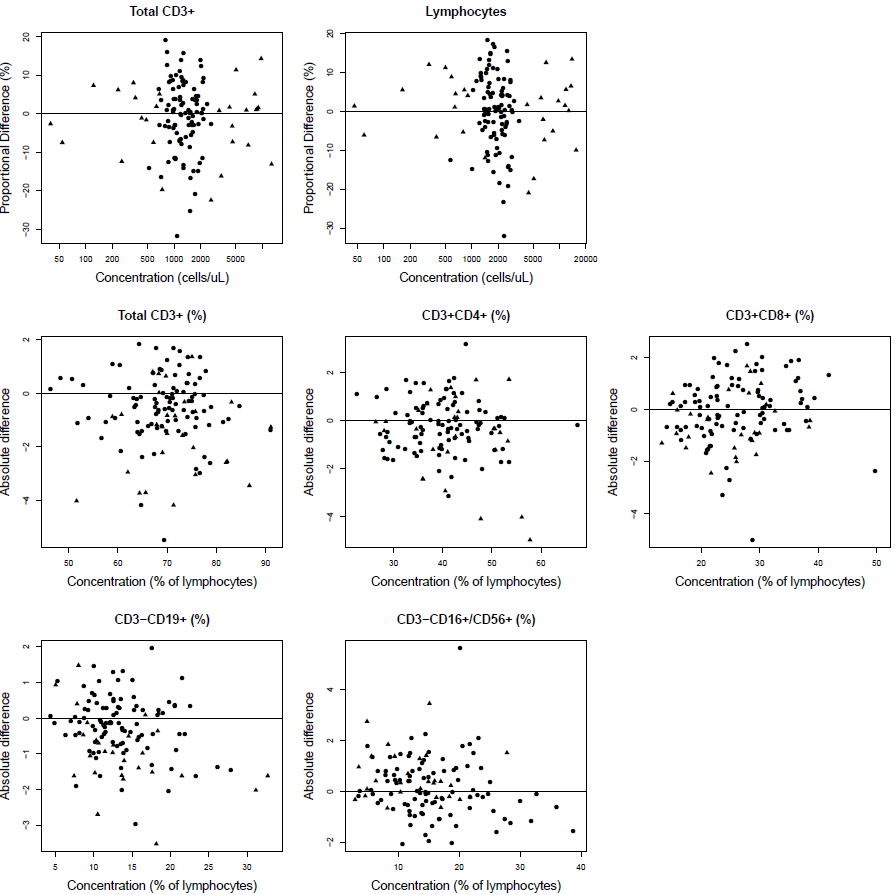


*Circles indicate native samples and triangles represent healthy samples in which leukocyte counts were either diluted or concentrated. X axes represent the mean concentration of the miniLab and comparator method results (Total CD3+, n = 116; remaining measurands, n = 123). Y axes represent the proportional or absolute difference of the miniLab results relative to the comparator results.

SUPPORTING INFORMATION FIGURE 4 Summary of the Zika virus assay workflow for the analytical sensitivity study.

SUPPORTING INFORMATION FIGURE 5 Summary of the anti-HSV-2 IgG assay workflows for the precision and method comparison studies.


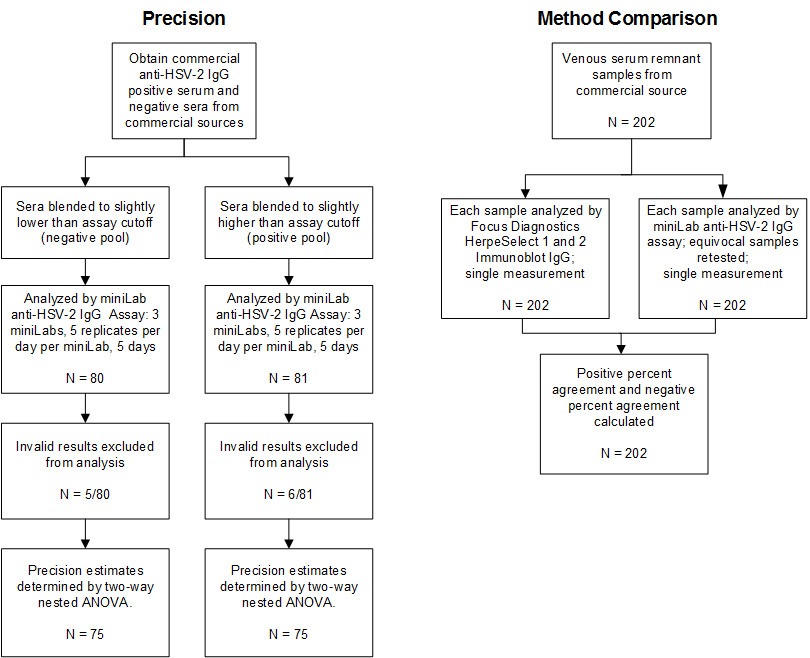


Abbreviations: ANOVA, analysis of variance; HSV-2, herpes simplex virus type 2; IgG, immunoglobulin G.

SUPPORTING INFORMATION FIGURE 6 Summary of the lipid panel workflows for the precision and method comparison studies.


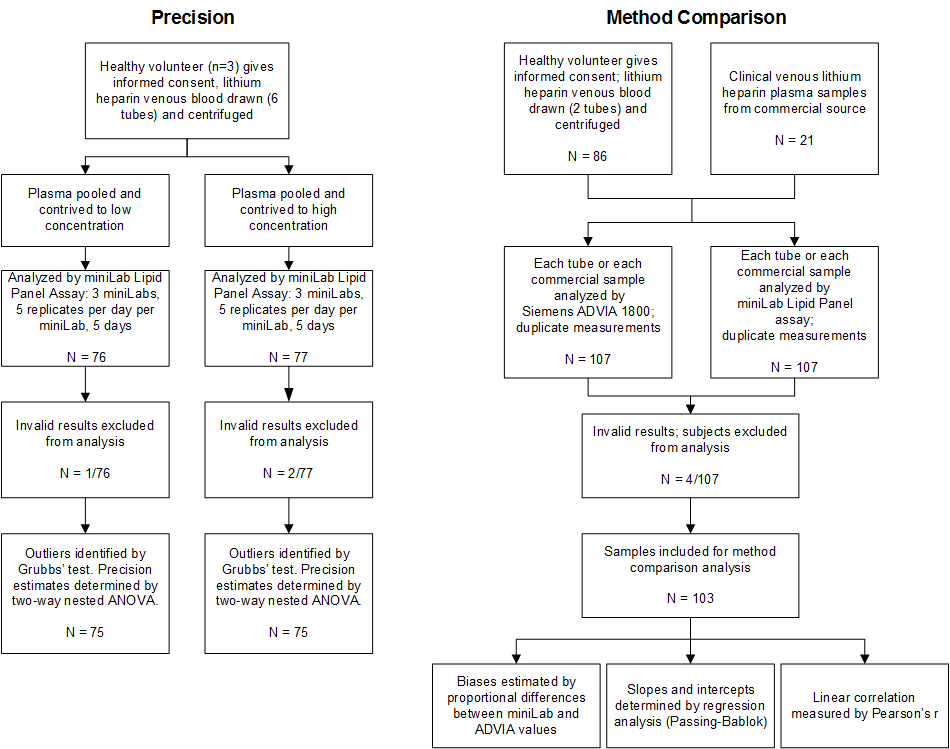


Abbreviations: ANOVA, analysis of variance.

SUPPORTING INFORMATION FIGURE 7 Summary of the lymphocyte subset panel workflows for the precision and method comparison studies.

Abbreviations: ANOVA, analysis of variance; EDTA, ethylenediaminetetraacetic acid.

SUPPORTING INFORMATION VIDEO 1 miniLab architecture and function. The layout of the miniLab and its modules are shown, and the miniLab performs example protocols representative of various analyte classes. Modules are shown at 0:50; hematology protocol at 2:20; clinical chemistry at 3:12; immunochemistry at 3:53; and molecular diagnostics at 5:43.
